# Breast Cancer Heterogeneity: A focus on Epigenetics and *In Vitro* 3D Model Systems

**DOI:** 10.22074/cellj.2018.5442

**Published:** 2018-05-28

**Authors:** Suresh Palamadai Krishnan

**Affiliations:** Department of Biomedical Sciences, School of Biosciences and Technology, Vellore Institute of Technology, Vellore, Tamil Nadu

**Keywords:** Breast Cancer, Epigenetics, Heterogeneity, *In Vitro*

## Abstract

Breast cancer (BC) is a widely prevalent form of neoplasia in women with fairly alarming mortality statistics. This aspect may be attributed, in part, to the current spatial and temporal heterogeneity-based limitations in therapies with possible recurrence of this tumour at primary and/or secondary sites. Such an extensive phenotypic heterogeneity in breast cancer is unlikely to be adequately or completely comprehended by an immuno-histopathology-based classification alone. This finding has warranted research and development in the area of microarray-based methods (i.e. transcriptomic and proteomic chips) for an improved molecular classification of this complex and heterogeneous tumour. Further, since epigenetics can also be an important determinant in terms of diagnosis, prognosis and therapy, this review provides an insight into the molecular portrait of BC in genetic and epigenetic terms. Specifically, the roles of characteristic DNA and histone-based modifications as well as mi-RNA- based alterations have been discussed with specific examples. Also, their involvement in epithelial mesenchymal transition (EMT) processes in cancer stem cells (CSCs) has been outlined. Last but not least, the salient aspects and the advantages of *ex vivo/in vitro* 3D model systems in recapitulating several aspects of BC tumour (particularly the architecture as well as the apico-basal polarity) are mentioned. This review hopes to provide not only an improved and updated understanding of the epigenetics of breast cancer, but to also elaborate on tumour model development/refinement, biomarker evaluation, drug resistance and test of individual drugs or drug combinations and drug delivery systems.

## Introduction

The most common site for the development of neoplasia in women is the breast. The mortality rate for those afflicted with this disease has been reported to be 25% (1). This statistic is alarming, despite the many advances in diagnosis and treatment. One of the major challenges in improving patient stratification, accurate prognosis and therapy optimisation is the complex, heterogeneous nature of breast cancer (BC) tumours (2). In this regard, the availability of public resources such as the cancer genome atlas (TCGA) provides an unprecedented opportunity to identify the molecular factors that would aid stratifying patients as responders versus those that are indolent to therapy. Also, improvements can be made in prognosis-based markers as well as in identifying markers for optimal therapeutic response. 

The TCGA repository of cancer-related molecular information was compiled following data generated at several omics levels including whole exome sequencing- based mutational spectra and DNA copy number changes, transcriptomic expression patterns (expression data) and reverse-phase protein array (RRPA)-related alterations (3). 

BC-related heterogeneity has been attributed, at least in part, to potentially reversible epigenetic alterations in the methylome and miRNA expression. Hence, a thorough characterization and analysis of the various epigenetic players ex vivo/ in vivo would provide an updated review of these molecules in human breast cancer tumours. However, a considerable amount of BC epigenetic data has been obtained from several model systems including *in vitro* assays. Hence, systematic cataloguing and updating of results obtained from *in vitro* models would serve as an extra piece of important information, provided the results are concordant with observations in humans or widely accepted *in vivo* xenograft animal models as well as transgenic animal systems. Furthermore, it is widely accepted that one of the major causes of treatment resistance, aggressive behaviour, disease severity, and rate of progression and relapse is due to cancer stem cells (CSCs) (4). 

Some or all of these cells may survive the currently employed treatment methods including chemo-and radiotherapy. This thus shows the need to evaluate and possibly refine existing model systems that can mirror, at least in part, the spatial and temporal epigenetic heterogeneity in breast CSCs. However, heterogeneity in the vasculature as well as the role of stromal factors is also an important determinant in mimicking the tumour in the context of its microenvironment. Hence, this review is to also provide an update with regards to the existing 3D model systems. 

This approach has been substantiated by several reports demonstrating that these systems are better alternatives to the 2D environment, wherein a monolayer of cells is cultured in artificial conditions. 

### Molecular features of breast cancer 

#### Ductal and lobular carcinoma

Based on the shape, structure and site of origin, breast tumours are broadly classified as invasive ductal carcinoma, not otherwise specified (IDC, NOS-more common) and ductal carcinoma in situ-ductal carcinoma in situ (DCIS). These tumours may be known as ductal or lobular based on the site of their origin. The term DCIS is a generic term that refers to the non-invasive, abnormal cell growth that is localized to the ducts and lobules and may become malignant. The IDCs refer to cancers that have infiltrated into the extracellular matrix region through the wall of the duct (2). 

A more recent and elaborate classification of BC has, however, changed IDC, NOS (2003) to invasive carcinoma of no special type (NST). The other types of BCs are of mixed types including invasive lobular carcinoma, tubular carcinoma and invasive cribriform carcinoma, carcinomas with medullary features, metaplastic carcinoma, carcinomas with apocrine differentiation, adenoid cystic carcinoma, mucinous carcinomas and carcinomas with signet-ring cell differentiation, invasive mucinous carcinoma, carcinomas with neuroendocrine features, invasive papillary and micropapillary carcinoma, secretory carcinoma, oncocytic carcinoma, polymorphous carcinoma, sebaceous carcinoma of the breast, lipid-rich carcinoma, glycogen-rich clear cell carcinoma, acinic cell carcinoma, microinvasive carcinoma and inflammatory carcinoma (5). 

Despite the similarities in the 5- and 10-year survival rates, the invasive lobular carcinoma (ILC) category of cancers (ER +ve, PR +ve and a HER2 +ve subset) is more likely to metastasize and bilateral variants are more frequent. Since the clinical course and the underlying biology are different, histology-based studies may not be sufficient for the accurate classification/sub-stratification of the five subtypes of ILC. Further, the non-IDC and non-ILC cancers (not listed above) include those that are comedo as well as the mucinous A (paucicellular) and B (hypercellular) subtypes. As is evident from the classification mentioned above, the challenges posed during BC therapy can be attributed largely to the complex and heterogeneous nature of the tumour (6).

In this context, the availability of RNA sequencing methods has provided the opportunity for a better classification based on the tumour transcriptome and for also identifying molecular mechanisms that may contribute to the observed variability in BC (e.g. exon skipping and promoter switching) (7). 

To circumvent this problem, molecular classification now differentiates ductal and lobular carcinomas into ER+/luminal (luminal A/luminal B), basal-like, Erb-B2+ and normal breast. These results were obtained following whole-transcriptomic microarray analysis of 40 breast tumours. These results were correlated with those obtained from 11 cultured cell lines. The genes selected were those showing a similar expression pattern in the same individual, while differences were observed among patients. 

Reproducibility of the results (independent and repeated sampling) as well as the strong correlation of the quantitative expression data between a primary and a metastatic tumour further added to the strength of the evidence. Hence, experimental designs of this nature (by analysing more genes) are warranted to refine the molecular signature and make it more representative of breast tumour variability (8). 

A similar hierarchical clustering approach was later used to stratify 115 malignant breast tumours using 534 "intrinsic" genes. This approach was replicated independently and confirmed the earlier findings and sub-stratified BC tumours into basal-like, two luminallike, normal-like and ERB-B2 over-expressing cancers. This approach was further validated by similar sub- stratification results reported in another two independent studies based on samples obtained from different patient populations. The selected patient groups tested were not only different in terms of age distribution, stage of the tumours, the microarray technology platforms utilised were also different. Nevertheless, variation was observed in gene clusters in the different populations studied. This level of variation may be due to the small number of genes studied, methodology-based variations and statistical testing issues. Another line of evidence for breast tumours being distinct entities was the increased formation of basal type BCs in individuals positive for *BRCA1*. In terms of clinical outcome, the basal and ERBB2+ subtypes are the most severe in terms of the period of time between primary tumour formation and metastasis, while the luminal B subtype is an intermediate between the two (9).

These results underscore the distinctiveness of the basal subtype and also the need to further classify the other subtypes in molecular terms, likely leading to a better diagnosis and prognosis. The luminal B subtype has been further characterized based on mutational analysis as well as methylation-based studies. *TP53, FOXO3* and *PIK3CA* genes were found to be frequently mutated. Other genes were found to be overexpressed, possibly due to copy number alterations (CNAs). Next-generation sequencing of another cluster of tumours have showed that *KCNB2*, *UTRN* (6q24) and *MDN1* (6q15) were mutated often in this subtype (10). 

The luminal A category tumours have been associatedwith specific mutations in *GATA3, PIK3CA* and *MAP3K1*. Furthermore, the existence of two categories of HER-enriched BC subtypes were identified accordingly (HER1, pHER1; HER2, pHER2) (3). This study added more information to the molecular portraitof these subtypes, which was originally describedby Perou et al. (8), and also provided evidence for heterogeneity and plasticity within rather than between subtypes. 

Amore recent paper has classified DCIS into two groups, namely DCIS-C1 and DCIS-C2. This classification was based on integrated pathway-based modelling analysis of RNA-sequence data. DCIS-C1 was classified as being representative of the more aggressive, highly proliferative, basal-like or ERBB2 subtype with characteristic features of a phenotype similar to that of Tregs. This subtype should be contrasted with DCIS-C2, in which tumours display a low to moderate proliferation capabilities. Furthermore, the DCIS-C2 tumour subtype was ER/PR double positive and luminal-like. In the context of epigenetic factors, lncRNA *HOTAIR* was upregulated, while the SOX family of tumour suppressor genes, particularly *SOX10, SOX11* and *SOX15* as well *HOXA5* was silenced (11). In the case of ILCs, the five major histological subtypes were classified into two categories based on their molecular architecture. The immune-related subtype showed an increase in the expression of *PD-L1, PD-1* and *CTLA-4* at the transcript level and exhibited a greater susceptibility to DNA-damaging agents in certain cell lines. The hormone-related subtype, was associated with epithelial mesenchymal transition (EMT) transitions, chromosomal gain (1q and 8q) and loss (11q) in addition to increase in the expression level (mRNA and the protein) of *PGR, ESR1, GATA3* and *FN1*) (12). 

#### Triple negative cancer molecular phenotype

The basal subtype of BC lacks ER, PR and HER2, and comprises approximately 16% of all BCs. This subtype is thus known as triple negative BC (TNBC), a phenotype that is recalcitrant to conventional therapies with an approximate 20% response rate. 

TNBC has a very poor prognosis with the medial survival being only 1 year and the relapse rate is about 30%. Hence, there is an imperative need to further stratify the TNBC subtype. Such an approach may provide a better understanding of the mechanisms involved in TNBC development as well as possibly introducing a better model for the development and/or refinement of ethno-based drugs (13).

Another triple negative subtype with a poor prognosis is known to be in the claudin-low category. This subtype of cells showed expression of EMT marker genes associated with immune response and stem cell-like features (14). Furthermore, these cells represent invasive ductal carcinomas with an excessive differentiation of medullary and metaplastic features apart from a low or absent expression of luminal differentiation markers. Interestingly, cell lines and animal models are available that mimic this subtype. These tools have received a lot of attention especially since the claudin-low sub-type closely mimics the BC stem cell and is an attractive epigenetic therapeutic target (15). 

In a gene expression analysis, 21 BC data sets comprising 587 TNBC cases were analysed, leading to the identification of six subtypes in addition to the identification of suitable model cell lines (for drug testing). The two basal-like subtypes (BL1 and BL2) had a higher induction of cell cycle and DNA damage response genes. The other subtype was immune-modulatory (IM), while the mesenchymal (M) and mesenchymal stem-like (MSL) subtypes had a molecular profile which resembled that of EMT transition. The luminal androgen receptor (LAR) subtype was linked to AR-mediated signalling and patients under this category included those that had a lower relapse-free survival rate (13). 

The heterogeneity seen in the tumours in vivo is mirrored largely in the commercially available BC cell lines. In cell line studies (MCF-7 versus MCF-7 derived cell lines), noncoding RNAs and possible differential splicing were identified apart from the presence of a cluster of genes whose expression was correlated to steroid-based drug response/lack of response (16). It is known that tumour heterogeneity (intra and inter tumoural) can be due to genetic and epigenetic factors, with these factors affecting differentiation and cell death (17). 

In specific, it has been shown that EMT processes, regulated transcriptionally and epigenetically, occur preferentially in BC cells with the basal-like phenotype. For instance, increased expression of markers that can be used to flag an EMT process (cadherin-11; N-cadherin; smooth muscle actin; vimentin) were observed. Also, an increased expression of ECM remodelling and invasion- related proteins (fascin, laminin and SPARC) was also reported. Last but not least, classical epithelial markers (E-cadherin and cytokeratins) were also shown to be down-regulated (18). 

This final aspect should be contrasted with an earlier report wherein high expression of membrane E-cadherin was linked to invasive carcinomas (with a pathology similar to that of a mixed ductal and lobular cancer), while its lack of expression was linked to lobular carcinoma. This study thus suggested E-cadherin membrane expression as a marker to distinguish between these two major histological subtypes of cancer (19).

#### Epigenetic processes and breast cancer

DNA hypo/hyper-methylation, histone acetylases and deacetylases, methyltransferases and demethylases are epigenetic changes considered to be important in BC. Micro-RNA-based alterations are also involved in terms of regulating epigenetic processes in BC.

Of note, PRC2 complexes are, in general, associated with gene repression, while PRC1 complexes are related to gene activation. Specifically, the PRC2 complex proteins like Suz12, EED and Ezh2 have been known to mediate gene silencing by the trimethylation of lysine 27 in the H3 histone proteins. The general concept is that euchromatisation ("open form") around the promoter region would favour accessibility of the transcription factors for the initiation of transcription. Heterochromatisation, however promotes a more repressed chromatin state and this alteration may thus be linked with the silencing of gene expression. Epigenetic regulation has been reported in diverse BC-related events including plasticity and EMT induction (20). The following section highlight the importance of key epigenetic events associated with neoplastic transformation of the human breast as well as BC stem cells.

### EZH2, EED, SUZ12 and tumourigenesis

The three proteins (EZH2, EED and SUZ12) are part of the PRC2 complex that is associated with gene silencing. Murine cell-based data as well as results from primary human tumours has provided evidence for the activation of Ezh2. This activation has been linked to certain upstream events involved in the pRb/E2F pathway. Deregulation of certain tumour suppressor genes (e.g., p16) results in the activation of the cyclindependent kinases (CDK4 and CDK6). 

This activation, in turn, hyper-phosphorylates pRb, thereby causing the release of E2F transcription factors to increased Ezh2 expression. EZH2 is known to methylate H3K27Me3 and H3K9Me3 (21), possibly in the regions upstream of tumour suppressor genes. This methylation thus provides a possible mechanistic link to gene silencing since HPC2, a protein member of the PRC1 complex, can be recruited to the PRC2 complex, thereby facilitating gene silencing.

### Involvement of H3K9Me3and H3K20 

It has been reported that desmocollin 3 (a cell-cell adhesion molecule in the family of cadherins) is down- regulated in certain BC specimens and is linked to aberrations in cytosine methylation in its promoter elements (22). MASPIN (a protease inhibitor associated with growth and metastasis in nude mice) may be inhibited by an epigenetic mechanism involving G9a (methyl transferase trimethylating H3K9Me3 at certain promoter regions), wherein cytosine methylation and hetero-chromatisation at the promoter region may account for its observed decreased expression which is associated with poor prognosis. Hence, G9a-mediated epigenetic regulation of MASPIN and other target genes may be considered as an important epigenetic factor (23).

Snail, one of the important transcription factors associated with the induction of EMT in primary tumour cells, it thought to recruit G9a. This protein is associated with *in vivo* recurrence of tumours and also a predictor of a decrease in relapse-free survival. The methylation of lysine 9 (H3K9Me2) in the region surrounding the E-cadherin promoter may contribute to the decreased expression of E-cadherin (24). 

A later study has further shown that Snail also recruits Suv39H1 (suppressor of variegation 3-9 homolog 1), a methyl transferase forming H3K9Me3 in the vicinity of the E-cadherin promoter, thus down-regulating its expression (25).

Furthermore, it has been shown that there is a correlative increase in the DNA methylation status (discussed in detail below) flanking the same gene, thereby providing evidence of a link between this type of epigenetic phenomena with that of key changes in histone proteins. 

This mechanism of Snail-mediated E-cadherin silencing is reiterated again in a more recent paper, in which certain histone methylation enzymes (PRC2, Suv39H1 and G9a) are recruited to contribute to the hetero-chromatisation in the vicinity of the E-cadherin promoter (H3K9Me3). The gene silencing then occurs due to the concomitant DNA hyper-methylation of the CpG islands. The DNA and histone-based epigenetic changes act in a coordinated manner to mediate EMT processes.

The following sequence of events occurs during the Snail-mediated choreography of multiple epigenetic events, culminating in the down-regulation of E-cadherin expression and in turn EMT processes. LSD1 (demethylase)/HDAC (deacetylase) is recruited to the E-cadherin promoter by Snail via its SNAG domain. This demethylase is involved in removing methyl groups from H3K4Me2/3, while the deacetylase removes acetyl groups from H3/H4. This demethylation event is thought to promote Snail-mediated recruitment of G9a and Suv39H1 on the E-cadherin promoter. HDAC may also be involved in aiding the interaction of Snail with PRC2, thereby contributing to the binding of the latter to the promoter elements. The sequential methylation of H3K9Me to H3K9Me2 and H3K9Me3 is mediated by G9a (interactions with the C-terminal domain of Snail) and Suv39H1 (via the SNAG domain of Snail) respectively. These two methyl transferases are also involved in the final step of E-cadherin down-regulation in recruiting DNA methyltransferases (DNMT) for hyper-methylationmediated silencing of E-cadherin, which is a significant event in EMT (26). 

There are other reports where G9a has been shown to act differentially in a cell type-specific manner. GATA3 forms a complex with G9a and NURD (MTA3) and silences ZEB2 (an important transcription factor involved in EMT induction). With BC progression, GATA3, G9a and MTA3 are down-regulated and ZEB2 is up-regulated. This upregulation, in turn contributes to the decrease in the expression levels of G9a and MTA3 via the G9a/ NURD (MTA1) complex (27). 

GATA3, a transcription factor involved in the mesenchymal epithelial transition (MET), plays a pivotal role in E-cadherin silencing via its transactivation domain. This event is mediated by the GATA-3-induced chromatin architecture changes (local histone modification and nucleosome eviction) or other changes that do not result in an accessible chromatin. Such MET requires binding as well as recruitment of BRG1, an ATPase of the SWI/ SNF family of chromatin remodelers (28). 

The SET8 (also known as PR-Set7/9, SETD8 or KMT5A) is a histone methyl transferase (HMT) with a SET domain. Its recruitment is mediated by TWIST (another transcription factor involved in the induction of EMT). This HMT, in turn, mono-methylates H4K20 and suppresses E-cadherin expression, while a similar methylation event in the N-cadherin promoter activates it (29). 

This apparently paradoxical finding can be explained based on the type of dimerisation of TWIST. In specific, homo-dimerization of TWIST leads to the activation of N-cadherin, however, hetero-dimerization of this transcription factor with Mi2/NuRD, MTA2, RbAp46, Mi2 and HDAC2 proteins leads to suppression by the formed complexes. This TWIST-mediated recruitment of the aforesaid complex proteins, to the promoter of E-cadherin, represses this gene, which is apart from direct binding of the transcription factor to the E-cadherin promoter (29, 30). 

### Involvement of H3K27Me3

A hydrolase inhibitor, 3-Deazaneplanocin A (DZNep), can cause the levels of ado-homocysteine to be elevated. This elevation, in turn, results in the decrease in the levels of EZH2, SUZ12 and EED. Hence, there is an inhibition of H3K27 and not H3K9 methylation marks, which leads to a reversible reactivation of certain genes. Significantly, activation of an effector of apoptosis (FBXO32) may account for DZNep-induced apoptosis in BC cells. This molecular baiss provides us an elegant approach for modulating epigenetic proteins that may possibly aid in selective activation of apoptosis in BC cells (31).

An increase in EZH2 is associated with increased invasiveness, increased proliferation rate and increased aggressiveness behaviour. It is also considered to be a marker for a pre-cancerous lesion in a tissue that is histologically normal (32). 

In both cases, EZH2-mediated trimethylation of H3K27Me3 leads to local heterochromatisation as well as DNMT1-mediated gene silencing. However, the final outcome may to some extent be dependent on the types of genes that are silenced (e.g. those that contribute to the neoplastic phenotype or those with pro-apoptotic function). Although Snail is involved in recruiting G9a to the H3K9 site, it has been reported that the activation of Snail is mediated by the removal of the repressive H3K27Me3 marks. These methylation marks are removed by the de-methylating action of KDM6B (also known as JMJD3–part of the Jumoji family) (33). 

The section below outlines the links between hypermethylation of the CpG islands in the promoter region and silencing of the gene while bearing in mind that multiple studies have reported DNMT1 and SNAIL1 to be involved in the repression of E-cadherin expression.

### H3K27 acetylation 

The p300-mediated acetylation (H3K27Ac mark), in addition to the repressive H3K27Me3 mark, is maintained by DUSP4, a phosphatase targeting threonine/serine and tyrosine residues. Knockdown of this phosphatase, as well as DUSP6, enhances stem cell formation, while down-regulation of DUSP1 decreases stem cell formation (CD44^hi^/CD24^lo^/EpCAM+breast CSCs). However, DUSP6 overexpression has been observed in HER2+ BCs. In this context, it is pertinent to point out that there are inhibitory and activating phosphorylation marks (1834 active and 89 inhibitory marks) that regulate the activity of the p300 HAT enzyme which can activate certain genes involved in the formation of euchromatin (34). 

### DNA hypo/hypermethylation

Hypomethylation and the possible consequent constitutive expression of the JAK/STAT pathway has been reported in CD44+/CD24 low putative BC stem cells. Also, increased expression of several genes associated with this pathway has been shown in the mammosphere model (35). 

Hypermethylation of cytosines in the regulatory region of the E-cadherin gene has been observed in an E-cadherin-negative BC cell line. Interestingly, treatment with a demethylating agent returns its expression (both transcript and protein) to normal levels, thereby providing evidence for this epigenetic modification being responsible for promoter inhibition. Furthermore, the expression of a reporter gene under the control of the E-cadherin promoter provided fairly definitive evidence of the presence of the transcriptional machinery in the E-cadherin-negative BC cells. In a later study, it was again demonstrated that E-cadherin methylation correlated with fibroblast- like morphology in BC cell lines. This phenotype also correlated with the expression of genes associated with EMT transition including TGF-ß-related genes and the genes involved in CDH1 regulation (*ZFHX1B* and *SNAI2* but not *SNAI1* and *TWIST*) (36). 

Specifically, epigenetic regulation in the form of DNA hyper-methylation and the consequent inactivation of the Wnt pathway (an important mitogenic cell signalling pathway) has been known to be involved in BC development. In this regard, Dickkopf2 (an endogenous inhibitor of Wnt signalling) can arrest cells in G0 and G1, and induce apoptosis. This study was undertaken on 10 BC cell lines, 98 primary tumours and 21 normal breast tissues (37).

Another study has reported that Dickkopf3 inhibited the canonical Wnt/β catenin pathway, thereby leading up to β-catenin migrating from the nucleus to the cytoplasm and the membrane. This mechanism along with the presence of reduced levels of active β-catenin can further activate the non-canonical JNK signalling. DKK3 was shown to inhibit BC cell migration due to a reversal of EMT and a decrease in stem cell markers (38). Recruitment of DNMT1 by δEF1 (ZEB1) may be associated with a decrease in E-cadherin expression due to hyper-methylation. This transcription factor, along with SIP1/ZEB2, is associated with the repression of E-cadherin expression, which is an important marker of the EMT phenotype characteristic of CSCs (39).

The ZEB1 transcription factor has been shown to be regulated by the asymmetric dimethylation of arginine 3 of histone H4 by PRMT1 (an arginine methyltransferase) at its promoter. This modification has been linked to EMT induction as well as senescence (40). While the concept of hyper-methylation and gene silencing is widely accepted, it is necessary to link this epigenetic change with alterations in microRNA (miRNA; small RNA molecules that are involved in gene regulation either by the degradation of target mRNA or by the inhibition of gene expression at the translational level) expression in tumours (41) with functional assays. Adoption of a battery of widely accepted assays can provide definitive or corroborative evidence for the involvement of certain miRNA in BC. The section below aims to provide an overview of the importance of miRNA in BC development. 

### miRNA and breast cancer

Hypermethylation at the miR-200c-141 locus and a concomitant increase in EMT features in an *in vitro* cellular model provided evidence for the simultaneous occurrences of these intermediate phenotypes. Specifically, the transcription factors ZEB1 and ZEB2 were up-regulated under these circumstances and may contribute to an increase in invasiveness and tumourigenicity (42). A negative correlation has been reported for the methylation status of the two promoters (P1 and P2) regulating the miR-200b gene. These results were observed in 8 out of 9 cell lines and *in vitro* reporter gene assays.

These results were substantiated in clinical samples, with hypermethylation at P1 linked to metastasis to the lymph nodes, while P2 showed association with loss of ER or PR. Results of this kind provide us a sound basis for validating and emphasizing the role of promoter hypermethylation at miRNA promoters as possible biomarkers for BC (43). For example, miR-18b, miR-103, miR-107 and miR-652 may be good predictors of an increased probability of tumour recurrence and reduced patient survival, and also serve as markers of prognosis in TNBC patients (44). Increase in the miR-30 expression has been linked to an increase in apoptosis possibly via its effects in down-regulating AVEN, an anti-apoptotic protein, in BT-IC cells grown under non-attachment conditions (45). 

A systematic step-wise experimental design involving a combination of microarray analysis, artificial neural network (ANN)-based data-mining, real-time PCR and correlation analysis with clinico-pathological features was followed. Seventy six differentially expressed miRNAs were identified by microarray analysis based on total RNA of blood samples from women with luminalA BC. The ANN-based strategy enabled the selection of 10 miRNAs (miR19b, miR-29a, miR-93, miR-181a, miR182, miR-223, miR-301a, miR-423-5p, miR-486-5 and miR-652) for follow-up. Of these, four of them may have biomarker potential, since they were down-regulated in affected women. In addition, the combined signature of three of these (miR-29a, miR-181a and miR-652) may discern tumours from controls (46). 

While the increased expression of ZEB1/2 transcription factors, mediated by the down-regulation of miR200, has been reiterated in basal-like BC, distal BC metastasis has been associated with an increase of this miRNA family. This implies a possible role for them in the establishment of these cancer cells at a site distal from its origin, which may possibly be due to a feed-forward loop-mediated repression of ZEB 1/2 by miR200 (47). 

It has been reported that the transcription of *PTPN6* and miR200c/141 are tightly linked together under a wide variety of physiological conditions. The regulation of miR200c/141 involves by-passing the expression of PTPN6 (SHP1) either by the use of an alternative polyadenylation signal or by a later termination of the transcription of *PTPN6* gene. The alternative mechanism may be based on DNA looping where the transcriptional machinery of both genes physically interact, providing an opportunity for a common epigenetic regulation (48). 

Moreover, miRNA regulate the behaviour of BC stem cells (BCSCs) (49). For instance, a miRNA signature has been developed that can predict the prognosis of BC in hormone receptor +ve, HER –ve BC patients. It can also classify these patients into high and low risk groups. This signature (based on miR-21, miR-30c, miR-181a, miR-181c, miR-125b, miR-7, miR-200a, miR-135b, miR-22 and miR-200c expression levels) also correlated with distant relapse-free survival (49). Irrespective of the miRNA profile, targeting genes linked to the CSC phenotype may currently be the approach of choice in epigenetic-based therapeutics. 

### Epigenetics and breast cancer stem cells 

Side-population BC stem cells expressing membrane-bound drug efflux transporters and other markers have been shown to contribute to the observed recalcitrance of the tumour to the drug as well as tumour recurrence. Also, these cells divided rapidly and exhibited a relatively high frequency of survival. BCSCs have been associated with the different stages of the multi-step process of BC including invasion and metastasis (50). 

A unifying model based on the presence of BC stem cells as well as the clonal evolution model has been proposed (51). This final model, based on the inherent plasticity of stem cells, includes the hierarchical aspects being different at different times and different regions of the tumour. This variability can be attributed to the internal and external pressures affecting the survival of the tumours. However, the heterogeneity associated with BC is also mirrored in the variable surface marker profile of BCSCs. This classification was done by comparing CSCs in the special histological type category with those observed in the non-special type category. 

Specifically, the CD44^+/high^, CD24^+/low^ cancer cells belong to the low grade, luminal subtype. The high grade (basal-like, claudin-low) subtype CSCs of the medullary, metaplastic cancer category exhibit the CD44+/CD24-/low/ALDH1+ CSC phenotype. This classification is important for diagnosis, prognosis and therapy (including the development of novel drug molecules) (51). Such markers may be used for cell isolation, monitoring treatment efficacy and diagnosis/ prognosis. 

### Methylation marks and cancer stem cells

There is a strong association between DNA hyper methylation and histone methylation-mediated loss of tumour suppressor gene expression. There is also an association between DNA hyper-methylation and histone deacetylation. KDM5B, a histone demethylase, acts on H3K4Me3 and its over-expression can repress cell proliferation, adhesion and migration. This demethylase acts in concert with NuRD and HDAC1 in contributing towards repression of genes associated with cell proliferation (52). 

DNMT1 expression has been associated with hypermethylation and suppression of ISL1 expression in mammary tumours as well as in BCSCs. Hence, down-regulation of DNMT1 and ISL1 may lead to a decrease in the population of stem cells. This axis may be useful for drug development (53). EMT transition (e.g., loss of DNA hypermethylation-mediated silencing of E-cadherin and activation of N-cadherin) and the epigenetic links to the loss of this stem cell feature is reiterated here to underscore its importance and its possible reversibility. However, such changes have to be measured at the population level rather than at the single cell level.

JARID1B (a H3K4 demethylase) expression was shown to be amplified in luminal breast tumours and is associated with an expression profile that is characteristic of this subset of cancers. High activity of this enzyme is also linked to poor outcome in patients (54).

### Epithelial mesenchymal transition, cell signalling and stem cells 

It is known that EMT transition has been consistently associated with CSCs in various cancers including BC. In all these cancers, epigenetic events may modulate the CSC phenotype and a number of examples with respect to this aspect are provided below. 

Apart from part/E2F and Ezh2, other signalling pathways such as Wnt/ß-catenin and RANK/RANKL have been associated with EMT induction in CSCs. Specifically, increase in RANK/RANKL signaling has been shown to increase the population of CD44+/ CD24- stem cells, and induce EMT and stemness in human mammary epithelial cells, thus being involved in BC tumour initiation, progression and metastasis (55). 

Studies have shown that Nodal signalling is associated with the aggressive features in BC. The endogenous negative regulator of Nodal (Lefty1-a regulatory protein normally sequestered in the hESC microenvironment) is not expressed in cancer cells (56), thereby providing a plausible mechanism for Lefty1-mediated epigenetic silencing-mediated uncontrolled growth of cancer cells (57).

The position-dependent effect of GATA-3 has been demonstrated by the observation that GATA-3 can alter the open versus closed state of chromatin at certain loci. At other positions, the sliding of the nucleosomes may not be associated with the formation of accessible chromatin. In addition, removal of the transactivating domain of GATA-3 can affect the reprogramming of chromatin without altering its binding ability (28). This may thus affect the formation of the MET phenotype. 

Despite the inherent complexities in mimicking the reported tumour heterogeneity, the ability to capture molecular changes *in vitro* has led to the development and/or refinement of 3D model systems. Such 3D model systems can help in validating the results obtained in terms of testing the apoptotic/anti-oxidant potential of ethno-derived biomolecules in 2D systems (58, 59). This type of analysis will enable us to better understand the strengths and limitations of the existing model systems and the rationale behind their development before validating novel findings in the classical xenograft/patient-derived xenograft model systems. This seems vital since it is widely accepted that the 2D environment does not fully recapitulate the complex interactions and the heterotypic signalling necessary to develop an adequate model system for mechanism-based research and drug testing. A catalogue of the important 3D BC model systems is presented in a tabular format below (Table 1). Also the major findings are indicated in terms of their ability to mimick, at least in part, the heterogeneity observed *in vivo*. 

Refinement of 3D models should take into account the heterogeneity in the vasculature and the role of the stroma (heterotypic signalling) as well as other spatial and temporal variation in the breast tumour. Accordingly, knowledge gleaned from research in the area of patient-derived xenografts would be extremely useful in terms of better understanding the mechanisms involved in BC patho-physiology as well as possibly providing a molecular basis for the often observed increase in drug resistance. Mounting evidence has shown that xenografts have a strong potential since they mimic the *in vivo* pathology of the primary tumour in terms of heterogeneity; behaviour and metastatic properties even after serial passaging. 

**Table 1:** 3D breast cancer model systems


Sl. No.	Details of the 3D model development	Key findings	Reference

1	Chamber slide well-initially coated with 100% Matrigel. After solidification (1mm in thickness), MCF-10A dispersed cells were plated on this gel. Medium had hormones, growth factors and 2% Matrigel. The assay medium was altered every 4 days. Cells grow and form clusters after 5-6 days in 3D culture, and subsequently form acini	Able to recapitulate and mimic many aspects pertaining to the architecture of the mammary gland (growth arrest and polarized acini)	(60)
2	A. 3D "embedded" assay-cells cultured by embedding in lrECM (soluble extract derived from the EHS mouse sarcoma cells)B. The 3D ‘on-top’ assay-cells are cultured on a dilute solution of lrECM. This cell suspension is placed gently on top of a thin lrECM gel	Formation of polarized, growth arrested acinus-like colonies-better mimics than 2D cultures-amenable to downstream processing of the molecules extracted from these cells cultured in 3D	(61)
3	The 3D 'on-top' assay to produce cells with different morphologies, namelyround mass, grape-like and stellate	"Signal transduction regulation" was found to be different in terms of gene expression profiles^*^ of cells grown in 2D versus 3D. Also, "Enzyme Regulator Activity" was also close to being statistically significant (in term of differences in the gene expression profile of the two systems.*It is expected that the differences would be greater since regulation can also occur post-transcriptionally (in the context of the gene expression).	(62)
4	5% Matrigel^T^^M^ drip was compared with 3D Matrigel^T^^M^drip with sECM and 5% ECM in terms of apico basal polarity. Also, it was examined whether collagen IV and/or laminin 111 is required for apical polarity.	5% Matrigel^T^^M^ drip with collagen IV is sufficient and necessary for establishing apico-basal polarity-a fundamental prerequisite for better understanding factors contributing to apical polarity loss (multilayer of cells and lack of basal positioning of the nuclei).	(63)
5	3D spheroid developed using SKBR-3 cells in a well pre-coated with HEMA (indicating the importance of the substratum)	HER2 homodimer formation favoured-signaling diverted from the PI3K/Akt pathway to the ERK 1/2, MAPK pathway. Homodimer a better target for trastuzumab. Phosphorylated PAK2 is part of the survival pathway since this protein is not inactivated by trastuzumab.	(64)
6	A co-culture model-3 major cell types- normal and malignant breast: luminal cells, myoepithelia cells and fibroblasts from the stroma (for the 1^s^^t^ time)	Organization into structures that reproduced features seen in the normal as well as that of the DCIS breast-homing of myo-epithelial cells around the luminal population-basement membrane disrupted; β4-integrin lost (as in DCIS in vivo) -importance of the tumour associated fibroblast; disrupted the co-unit organization	(65)
7	This type of model mimics the structural and functional aspects of normal and malignant breast cancer tissues. MCF-10A cells were suspended in a collagen gel. These cells formed both acinar and tubular structures. The gel should be detached well from the cell culture plate. Cell contraction should occur in the suspension stage.	Collagen organization as well as biomechanical factors (cell-collagen interactions) is important for formation, elongation and branching of ducts.	(66)
8	Microscale cavities were created in the type I collage gel mould. This was done using certain posts with a defined geometry and spacing. Epithelial cells were seeded into these cavities and another layer of collagen was placed over the cells.	Depending on the shape of the cavities, hollow tissues were formed. Morphogenesis was observed after 1-3 days of culture. This experimental design can be extended to study interactions between luminal epithelial and myoepithelial cells.	(67)
9	Patient-derived mammary epithelial cells (reduction mammoplasty-cell suspension triturated, washed and depleted of fibroblasts) were used for the 3D culture using a hydrogel with defined components (collagen I, hyaluronan, fibronectin and laminin).	Under these defined experimental, serum-free conditions, the cells were converted into a morphological complex structure mimicking the native breast tissue (in terms of a central lumen, formation of lipid droplets, similar ductal morphology and branching). This branching commenced from a cluster of cells that expressed putative mammary stem cell markers	(68)
10	Myo-epithelial and luminal cells (from reduction mammoplasty)were combined in a collagen gel matrix. The structure formed was a physiologically relevant surrogate of the in vivo bilayer structure. Furthermore, induction of HER2 expression selectively in the luminal compartment may lead to the filling of the luminal cavity	This experimental design demonstrates the importance of the collagen matrix as well as the roles of the two cell types. This 3D model mimics, at least in part, DCIS. Hence, this model may be used as a testing tool for drugs/biopharmaceuticals targeting HER2	(69)
11	MCF-7 cells were cultured under 3D conditions using calcium alginate hydrogel. The proliferation rate correlated with the elastic modulus of the gel.	Under 3D conditions, the cells formed spheroids with their conformation similar to what is observed in vivo. The maximal proliferation rate was measured after 2 weeks for the softest hydrogel (E=150-200 kPa). This approach may be used as a tool to develop a more relevant model for in vitro cancer studies.	(70)


## Conclusion

The use of cutting-edge molecular tools has provided a better molecular portrait (based on genetic and epigenetic features) of BC that correlate with several variables including biological characteristics, diversity, clinical course and patient outcome. Comparative analyses of the epigenetic molecules, including those related to CSCs and EMT processes, in different tissues may facilitate the development and/or refinement of the existing signatures. This approach may not only aid the development and/or validation of the existing 3D model systems that better resemble the tumour phenotype, but it may also validate cell line-based and patient-derived xenografts. This would eventually lead to an improved understanding of the underlying mechanism and a better predictive power in terms of biomarker development, clinical course, response to drugs and drug combinations (from natural or synthetic sources) in addition to elucidating an epigenetic basis for the acquisition and maintenance of drug resistance. 
